# Inducing Drought Resilience in Maize Through Encapsulated Bacteria: Physiological and Biochemical Adaptations

**DOI:** 10.3390/plants14050812

**Published:** 2025-03-05

**Authors:** Tiago Lopes, Pedro Costa, Paulo Cardoso, José Almeida e Silva, Etelvina Figueira

**Affiliations:** 1 Department of Biology, University of Aveiro, 3810-193 Aveiro, Portugal; tslopes@ua.pt (T.L.); pedrommrscosta@ua.pt (P.C.); pjcardoso@ua.pt (P.C.); 2 CESAM—Centre for Environmental and Marine Studies, University of Aveiro, 3810-193 Aveiro, Portugal; 3 Sociedade Agrícola da Mumba, S.A., Menongue, Cuando Cubango, Angola; almeidasilva@omatapalo.com

**Keywords:** osmotolerant rhizobacteria, drought, starch-alginate capsules, antioxidant and physiological mechanisms

## Abstract

Droughts are projected to become prevalent throughout the 21st century, endangering agricultural productivity and global food security. To address these challenges, novel strategies to enhance water management and augment plant resilience are imperative. Bacterial encapsulation has emerged as a promising approach, offering benefits such as enhanced bacterial survival, soil compatibility, and sustainable plant growth. This study evaluated the osmotolerance of bacteria from arid environments and determined their plant growth-promoting ability in drought conditions. The encapsulation of these bacteria in bio-compatible capsules led to a substantial enhancement in the performance of maize plants under drought stress. Maize plants treated with encapsulated bacteria demonstrated a 35% increase in root biomass and a 28% enhancement in shoot growth compared to untreated controls. Furthermore, significant physiological and biochemical adaptations were observed, including a 45% increase in photosynthetic pigment concentration and higher osmolyte levels, which contributed to improved drought stress tolerance. The findings of this study demonstrate the potential of encapsulated bacteria to enhance maize resilience to drought, thereby supporting robust growth under water-limited conditions. This approach presents a sustainable strategy to improve drought tolerance, and it may reduce irrigation dependency and maintain crop yields in the face of increasing climate uncertainty.

## 1. Introduction

Climate change is altering weather patterns, modifying the annual rainfall, increasing its irregularity, and the frequency of extreme rainfall events, which, combined with increased temperature, contributes to more prolonged and severe droughts [1]. As a result, agricultural activity in drought-affected regions is likely to experience reduced crop yields [2,3,4], particularly for staple crops such as maize [5], wheat [6] and soybeans [7]. New sustainable water management strategies are being implemented to reduce the amount of water used in agriculture while maintaining productivity. The breeding of drought-tolerant crop varieties, the use of organic mulching and plastic films, and irrigation practices such as drip, micro-sprinkler, or overhead irrigation systems are currently used to maintain productivity with increased water use efficiency [8]. Feeding a constantly growing population has been achieved in recent decades using inorganic fertilizers and agrochemicals with harmful environmental consequences. Thus, there is an urgent need for new solutions that provide adequate crop nutrition and high yields with less impact on the environment, which can also increase crop resilience to drought while promoting soil health and fertility.

The use of plant growth-promoting rhizobacteria (PGPR) as biostimulants to enhance plant growth and resilience to biotic and abiotic stresses is emerging as a sustainable alternative to conventional methods [9]. These bacteria enhance nutrient availability through nitrogen fixation, phosphate and potassium solubilization, and iron sequestration [10,11], producing and modulating phytohormone synthesis, including indoleacetic acid, cytokinin, and gibberellins [12], and compatible solutes such as soluble sugars, proline, and alginate [13]. In addition, PGPR produce volatile organic compounds (VOCs) that induce specific plant responses (the quenching of reactive oxygen species (ROS), stabilizing cell membranes, activating specific metabolic pathways), facilitating plant growth in nutrient-poor soils, and increasing resilience to different stresses [14,15].

Introducing new bacteria into soils can be particularly challenging because of intense competition from indigenous microflora, unfavorable physicochemical conditions (e.g., pH and temperature), and significant fluctuations, which can affect the survival of the new bacteria introduced and the efficiency of plant-bacteria interactions [16]. Alginate, a naturally occurring polysaccharide extracted from brown seaweed, is both biocompatible and biodegradable. It forms a gel matrix with the controlled release of cells and compounds, making it an exceptional encapsulating agent [17]. In the context of PGPR encapsulation, sodium alginate provides a protective environment that enhances the viability and stability of these beneficial microorganisms under adverse environmental conditions [16,18]. The controlled release of PGPR offered by encapsulation improves root colonization and plant growth [19]. In addition, the gel-forming properties of sodium alginate enable the formation of capsules that can be conveniently applied to seeds or soil, thereby increasing the efficiency of PGPR and making this technique very attractive for agricultural applications [20,21].

Alginate matrices tend to lose integrity over time, and, when rehydrated, the viability and slow release of bacteria into the environment can be compromised [22]. Incorporating additives such as starch, clay, humic acid, skim milk, and sugars into the encapsulation process can help maintain the bacterial stability within the capsules, preserve the integrity of the capsules, and improve their adaptability to field conditions [23,24]. Starch is an excellent encapsulation additive. It is inexpensive and enhances capsule resistance to physical stress by increasing capsule viscosity, reducing porosity, and allowing the slow release of bacteria into the environment [22,24]. Additionally, some microorganisms can utilize starch as a carbon source, and adhesion to starch granules helps maintain a stable microbial population during preparation and storage [25].

Given the advantages of PGPR and encapsulation techniques, this study aimed to evaluate the effects of different encapsulated bacteria on maize growth under drought stress conditions. To achieve this goal, we characterized the osmotolerance and plant growth-promoting (PGP) properties (indole acetic acid, proline, alginate, P solubilization, K solubilization, siderophores) of bacteria that have been isolated from plant roots under dry conditions. Subsequently, a selected set of bacteria with the best osmotolerance and PGP trait properties were used for *in planta* testing to screen for the best bacterial strains to increase the resilience of plants subjected to drought stress. Strains with the highest ability to enhance plant growth were selected for encapsulation. Starch–alginate capsules containing the selected bacterial strains were used in a second *in planta* experiment to evaluate the effect of encapsulated bacteria on plant tolerance to drought by determining changes in plant biometrics and biochemical and physiological status.

## 2. Results

### 2.1. Bacteria Selection

**Figure 1:**
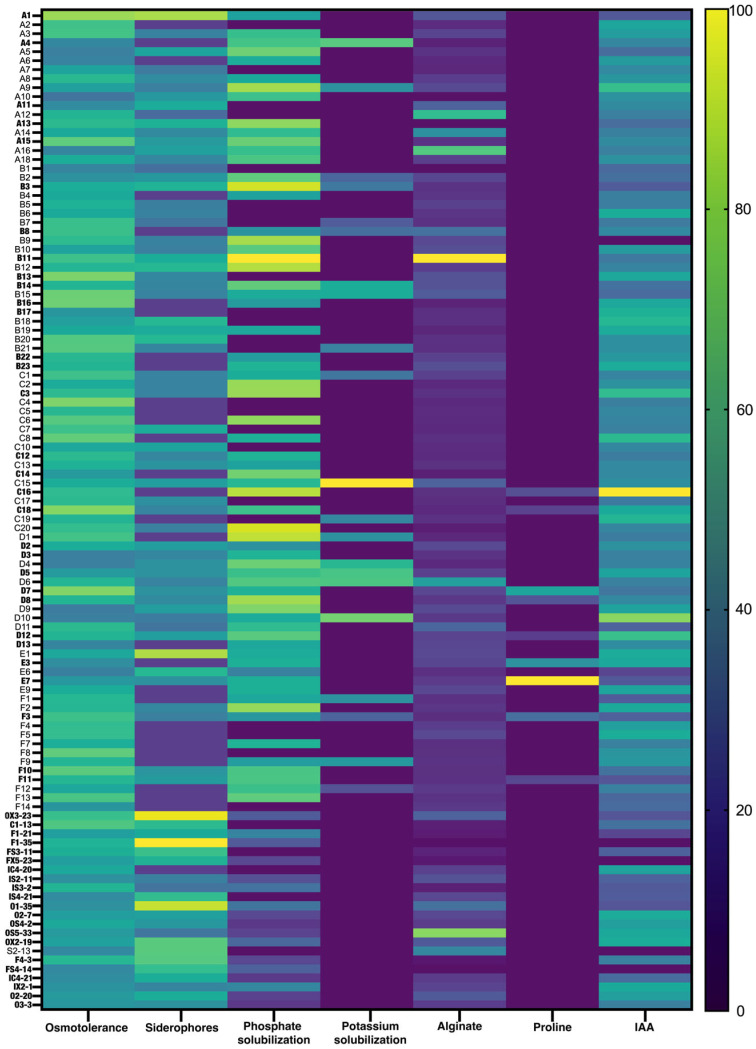
**Osmotolerance and plant growth promotion traits** (siderophore production, phosphate and potassium solubilization, alginate, proline, and indole-3-acetic acid synthesis) of bacteria isolated from the roots of plants grown in arid environments. Values represent the mean of three replicates per strain. Codes representing bacterial strains selected for subsequent work are marked in bold.

The osmotolerance and plant growth promotion traits (indole-3-acetic acid, siderophores, phosphate, and potassium solubilization) and drought tolerance traits (alginate and proline) of the bacterial strains are depicted in a heatmap (Figure 1). The majority of the strains presented medium osmotolerance, with an IC50 between 9% and 13%, but some displayed higher osmotolerance (>15%). Three isolates (C4, C18, and D7) displayed IC50 values of 21, 22, and 22.8%, respectively.

Most bacteria were able to produce siderophores, solubilize phosphate, and produce alginate. However, only a few (12%) could solubilize potassium and produce proline and indole-3-acetic acid under osmotic stress (10% PEG). With this in mind, 52 isolates were selected from the 118 evaluated. Priority was given to the osmotolerance levels; strains selected showed osmotolerance levels between 15 and 22.8% and higher PGP trait ability.

**Figure 2:**
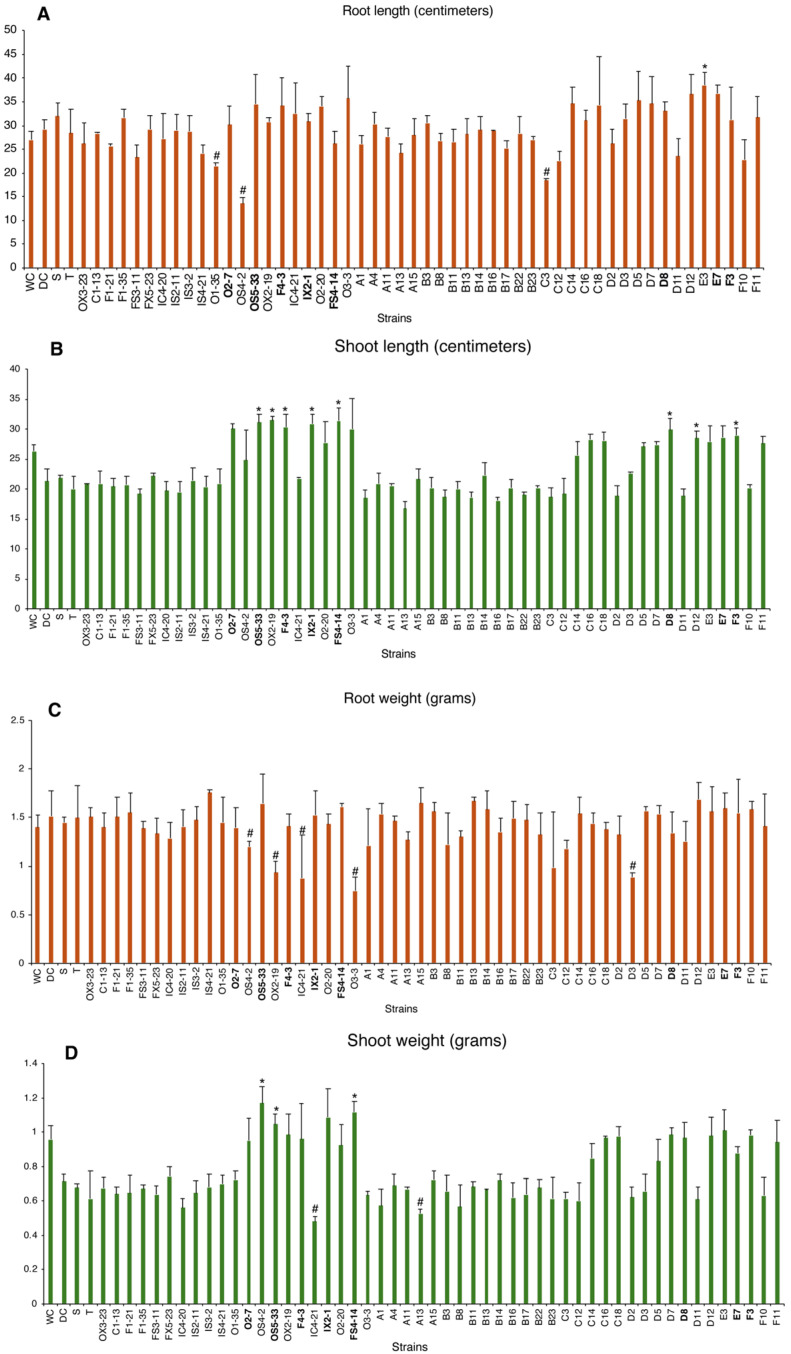
**Bacteria screening for the capacity to improve plant tolerance to drought.** Maize plants are either grown under drought conditions (35% water hold capacity (WHC) or not (DC). A watered control (60% WHC)—WC was also included. (**A**) Root length (cm); (**B**) Shoot length (cm); (**C**) Root weight (g); (**D**) Shoot weight (g). Values are means of at least 5 replicates, and error bars represent standard deviation. Asterisk (*) and cardinal (#) indicate significantly higher and lower values (*p* < 0.05), respectively, compared to the drought control (DC).

The 52 isolates (displayed in bold in Figure 1) were selected for a new round of screening (plant growth promotion under drought stress conditions). The influence of bacteria on maize plants grown under drought stress is presented in Figure 2. Drought decreased shoot length and weight and increased root length and weight. Bacterial inoculation displayed different results on shoots and roots, with some increasing and others decreasing growth. Eight strains showed significantly increased shoot growth and were selected for subsequent work (bold caption in Figure 2). The strains selected belong to the genera: E7: Unknown; OS5-33: *Pseudomonas*; O2-7: Unknown; F4-3: Unknown; D8: *Pseudomonas*; FS4-14: *Acinetobacter*; IX2-1: *Pantoea*; and F3: *Rhizobium*.

### 2.2. Influence of Inoculated Capsules on Maize Tolerance to Drought

**Figure 3:**
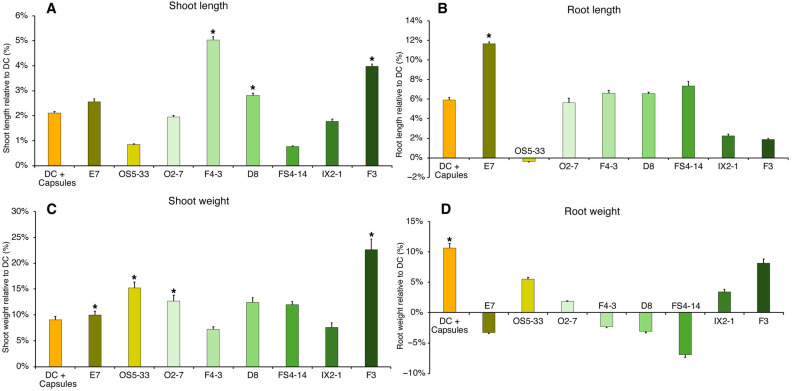
Effect of encapsulating the pre-selected bacteria with the best PGP and plant growth promoting capacity under drought conditions. Root and shoot length (**A**,**B**) and weight (**C**,**D**) relative to the drought control (DC) of maize plants under drought conditions (35% WHC) and exposed to different encapsulated bacterial strains and capsules without bacteria (DC + capsules). Values are means of at least 5 replicates, and error bars represent standard deviation. Asterisk (*) indicates significant differences (*p* < 0.05) between conditions and DC.

The uninoculated capsules positively affected the root growth (Figure 3B,D) of drought-stressed plants but only significantly so for root weight (Figure 3D). Most encapsulated bacteria promoted root length (Figure 3B), which was only significant for E7 strain. Root weight (Figure 3D) was not significantly altered by encapsulated bacteria compared to drought control (DC), although some strains (OS5-33, IX2-1, and F3) were able to increase root weight (4 to 8%) compared to DC.

In shoots exposed to drought conditions, non-inoculated capsules increased the shoot length (Figure 3A), and weight (Figure 3C); however, the difference was not statistically significant. Encapsulated bacteria increased shoot length (1–5%) compared to DC, with strains F4-3, D8, and F3 significantly increasing shoot length. All encapsulated bacteria positively affected shoot weight (4–22%), and E7, OS5-33, O2-7, and F3 significantly increased shoot weight.

### 2.3. Biochemical Alterations in Roots

#### 2.3.1. Oxidative Damage (Lipid Peroxidation and Protein Carbonylation)

**Figure 4:**
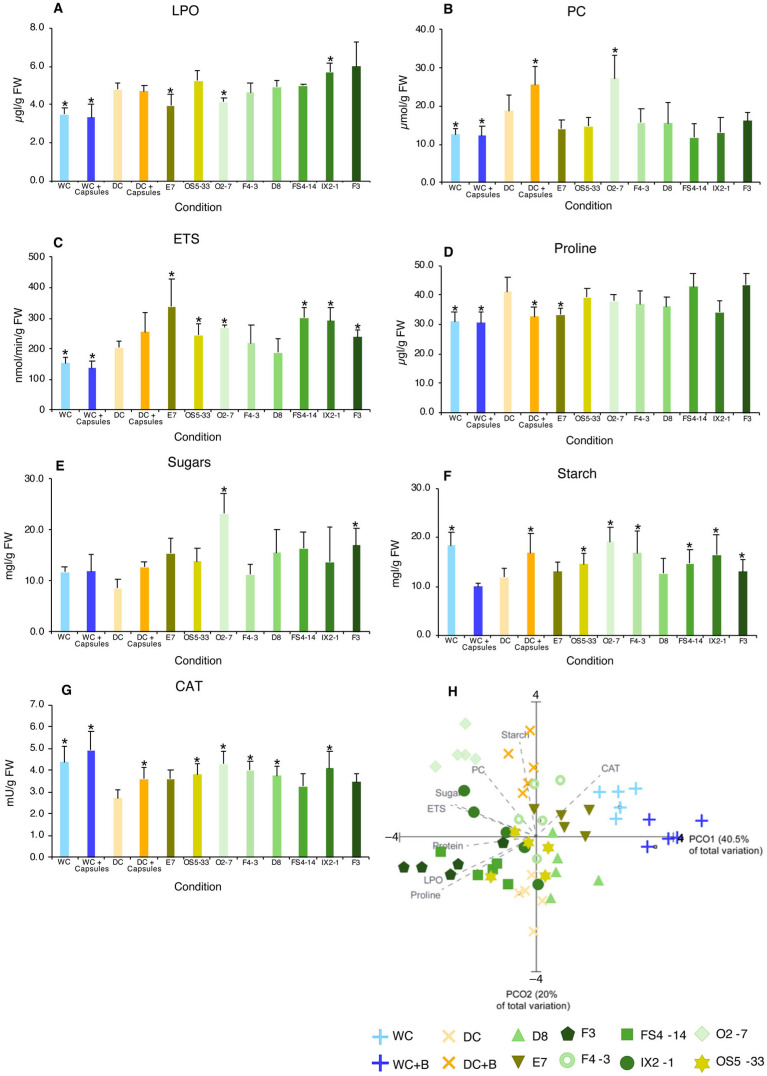
**Biochemical parameters of roots from maize plants grown under different conditions**: WC—watered control (60% WHC); WC + capsules—watered (60% WHC) + non-inoculated capsules; DC—drought control (35% WHC); DC + capsules—drought (35% WHC) + non-inoculated capsules; and drought (35% WHC) + inoculated capsules with one bacterial strain (E7, OS5-33; O2-7, F4-3, D8, FS4-14, IX2-1, F3). (**A**) LPO—lipid peroxidation; (**B**) PC—protein carbonylation; (**C**) ETS—electron transport system activity; (**D**) proline content; (**E**) sugar content; (**F**) starch content; (**G**) CAT—catalase activity; and (**H**) Principal Coordinate Ordination (PCO) of biochemical parameters (r ≥ 0.70). Values are means of at least 5 replicates, and error bars represent standard deviation. Asterisks (*) indicate significant differences (*p* < 0.05) between the different conditions with drought control (DC).

Under drought and non-inoculated conditions (DC), lipid peroxidation (LPO) (Figure 4A) was significantly higher than in watered conditions (WC and WC + capsules). The presence of non-inoculated capsules showed no difference from that in the DC condition. The addition of inoculated capsules resulted in different results. Strains E7 and O2-7 significantly reduced LPO levels compared to DC. Most strains did not significantly change LPO levels from DC, while strains IX2-1 and F3 increased LPO significantly for IX2-1.

Protein carbonylation (PC) (Figure 4B) was also higher under drought (DC) than under watered conditions (WC and WC + capsules). The presence of non-inoculated capsules significantly increased the PC levels relative to DC, and a similar and significant increase was observed in the O2-7 strain. The other encapsulated strains showed decreased PC relative to DC, although the difference was not significant.

#### 2.3.2. Metabolic Capacity (ETS) and Energy Reserve (Starch) Content

Drought (DC) significantly increased the activity of the electron transport system (ETS) (Figure 4C) than under watered conditions (WC and WC + Capsules). Non-inoculated capsules showed increased ETS activity compared with DC, although the difference was not significant. The addition of inoculated capsules generally increased ETS activity compared with DC, which was significantly higher for most strains (E7, OS5-33, O2-7, FS4-14, IX2-1, and F3).

The starch content (Figure 4F) decreased in DC compared to that in WC, although this decrease was not statistically significant. The presence of non-inoculated capsules significantly increased the starch content in roots compared to that in DC. Exposure to inoculated capsules increased starch content, with significant increases in the OS5-33, O2-7, F4-3, FS4-14, IX2-1, and F3 strains.

#### 2.3.3. Osmolyte Content

Drought (DC) increased the proline levels (Figure 4D) more than under watered conditions (WC and WC + capsules). The presence of non-inoculated capsules significantly decreased proline levels compared to those in DC. Exposure to the inoculated capsules resulted in small changes compared with DC. A significant reduction was observed in E7.

Drought stress did not induce significant differences in the sugar content (Figure 4E). The presence of inoculated and uninoculated capsules generally increased sugar levels, which was significant for the encapsulated O2-7 and F3 strains.

#### 2.3.4. Antioxidant Enzymes (CAT and SOD)

Catalase (CAT) activity (Figure 4G) was significantly decreased by drought. The presence of non-inoculated capsules resulted in a significant increase in CAT activity compared with that of DC. CAT activity was also higher in inoculated capsules than in DC but not significantly for some strains and significantly for others (E7, FS4-14, and F3).

SOD activity varied between 4.47 and 5.75 U/g FW and was not significantly different among the conditions tested, and results were not presented.

#### 2.3.5. Principal Coordinate Ordination (PCO) Analysis

From the multivariate analysis (Figure 4H) of root biochemical changes induced by drought and the addition of capsules (inoculated or not), it can be observed that PCO1 explained 40.5% of the total variation. This axis separated watered conditions (WC and WC + Capsules) on the positive side from drought conditions (DC and DC + capsules) on the negative side of the axis, indicating that drought was the main factor of variation among the conditions. The addition of inoculated capsules induced biochemical changes in the roots, with the main biomarkers as proline, LPO, sugars, and the ETS. This evidence suggests that the presence of some bacterial strains (FS4-14 and F3) increases the synthesis of osmolytes (proline), soluble protein, and membrane damage (LPO). Other bacteria (O2-7 and IX2-1) induced plant metabolic rate (ETS) and sugar levels, as well as protein damage (PC).

### 2.4. Biochemical Alterations in Shoots

#### 2.4.1. Photosynthetic Pigments (Chlorophylls a and b and Carotenoids)

**Figure 5:**
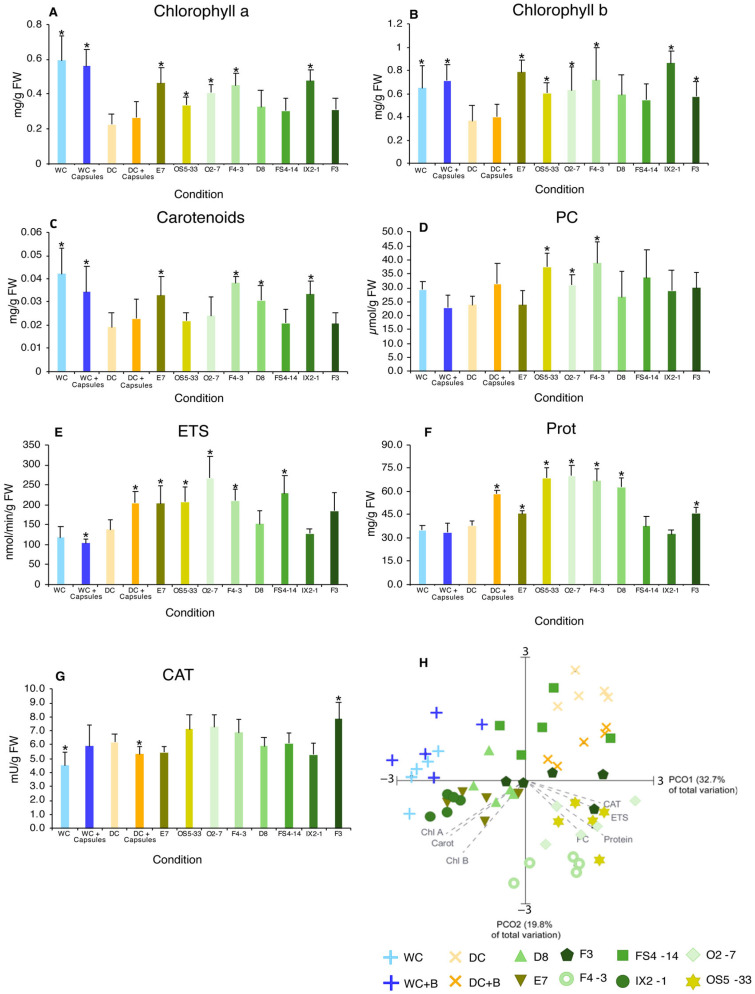
**Biochemical parameters of shoots from maize plants grown under different conditions** (WC—watered control (60% WHC); WC + capsules—watered (60% WHC) + non-inoculated capsules; DC—drought control (35% WHC); DC + capsules: drought (35% WHC) + non-inoculated capsules; and conditions containing selected bacteria: drought (35% WHC) + inoculated capsules with bacteria). (**A**) Chlorophyll a content; (**B**) chlorophyll b content; (**C**) carotenoid content; (**D**) PC—protein carbonylation; (**E**) ETS—electron transport system activity; (**F**) Prot—protein content; (**G**) CAT—catalase activity; and (**H**) Principal Coordinate Ordination (PCO) of biochemical parameters (r ≥ 0.70). Values are means of at least 5 replicates, and error bars represent standard deviation. Asterisks (*) indicate significant differences (*p* < 0.05) between the different conditions with drought control (DC).

Drought (DC) significantly reduced the photosynthetic pigments (Figure 5A–C) compared with the watered conditions (WC and WC + capsules). Non-inoculated capsules did not influence the chlorophyll a, chlorophyll b, and carotenoid contents. Conversely, exposure to encapsulated bacteria increased the levels of the three pigments compared to DC, with strains E7, OS5-33, O2-7, F4-3, and IX2-1 that significantly increased chlorophyll and carotenoid contents compared to DC.

#### 2.4.2. Oxidative Damage (PC and LPO)

Protein carbonylation (PC) (Figure 5D) decreased in drought (DC) compared to that in watered plants (WC), although not significantly. The presence of non-inoculated capsules increased PC levels compared with DC, although the difference was not significant. Exposure to inoculated capsules also significantly increased PC levels compared to DC for OS5-33, O2-7, and F4-3.

LPO levels varied between 5.17 and 7.44 nmol/g FW and were not significantly different among the conditions tested.

#### 2.4.3. Metabolic Capacity (ETS and Protein)

ETS (Figure 5E) activity did not change significantly due to water availability (DC compared to WC). However, non-inoculated capsules changed this scenario, with drought (DC + capsules) inducing significantly higher ETS activity than in the watered condition (WC + capsules). Exposure to inoculated capsules significantly increased ETS activity for most strains compared to DC for E7, OS5-33, O2-7, F4-3, and FS4-14 strains.

The protein content (Figure 5F) was similar among the DC, WC, and WC + capsule conditions. The addiction of non-inoculated capsules under drought (DC + capsules) significantly increased protein content compared to DC. The presence of inoculated capsules also significantly increased protein content, in most conditions (E7, OS5-33, O2-7, F4-3, D8 and F3) when compared to the DC.

#### 2.4.4. Antioxidant Enzymes (CAT and SOD)

Drought increased catalase (CAT) activity compared to WC but not WC + capsules (Figure 5G); however, adding non-inoculated capsules significantly decreased CAT activity (DC + capsules compared to DC). Inoculated capsules displayed different responses, with some strains showing increasing (F3, significantly) and others showing decreasing (not significantly) CAT activity.

SOD activity varied between 4.18 and 6.33 U/g FW and was not significantly different among the conditions tested, and results were not presented.

#### 2.4.5. Principal Coordinate Ordination (PCO) Analysis

From the multivariate analysis (Figure 5H) of the shoot biochemical status among the tested conditions, it was observed that PCO1 explained 32.7% of the total variation. This axis separated the watered conditions (WC and WC + capsules) on the negative side of the axis from the drought conditions (DC and DC + capsules) on the positive side, indicating that drought was the main driver of variation among the tested conditions. PCO2 accounted for 19.8% of the total variation and reflected the different effects of encapsulated bacteria on shoot biochemistry. The main biomarkers are photosynthetic pigments (Chl. a and b and carotenoids), the ETS, CAT, and protein. The presence of E7, D8, and IX2-1 was highly correlated with photosynthetic pigments (chlorophylls a, b, and carotenoids), bringing these conditions closer to watered conditions. On the other hand, strains O2-7, F4-3, and OS5-33 were more strongly correlated with protein, the ETS, CAT, and PC.

## 3. Discussion

The present study investigated the possibility of increasing plant tolerance to drought by adding capsules containing plant growth-promoting osmotolerant bacteria to soil. For this purpose, a three-phase selection process was adopted. The first phase included the isolation of bacteria from the root system of plants growing in locations subject to frequent droughts, as is the case for legume species growing in natural areas in southern Portugal, where summers are hot and dry, and natural vegetation from locations that are extremely dry year-round, such as the Namib Desert in Angola.

This strategy was effective in the second screening phase, since all strains presented an IC50 ≥ 9% PEG, indicating no osmo-sensitive strains [13]. Furthermore, 41% of the strains had IC50 > 15% PEG and were classified by the same authors [13] as moderately tolerant or tolerant to osmotic stress; three strains from this group were found to be extremely osmotolerant (IC50 ≈ 22% PEG). At this stage, 52 strains (48%) were selected for the next phase, considering their osmotolerance, plant growth promotion, and tolerance abilities. In the third phase, drought-stressed plants were inoculated with each of the 52 strains, and their ability to promote growth under drought conditions was assessed. Most strains showed increased growth, corroborating that osmotolerant strains positively affected plant tolerance to drought [26,27,28,29]. Eight strains (OS5-33, OX2-19, F4-3, IX2-1, FS4-14, D8, E3, and F3) were able to significantly increase the growth of plants experiencing drought stress. Some of these strains belong to well-characterized bacterial genera, reported to exhibit PGP traits and plant growth promotion under drought. The genus *Pseudomonas* is already known for root colonization, the production of exopolysaccharides, siderophores and phytohormones [30]. The *Rhizobium* genus is able to fix N_2_ and to produce beneficial traits that regulate plant growth, including phytohormones. [31]. *Pantoea* and *Acinetobacter* genera have already been described as plant growth inducers [32,33].

The growth of plants under drought conditions and inoculated with the eight bacterial encapsuled strains confirmed an increase in growth compared to drought-stressed non-inoculated plants, but not all strains could significantly induce plant growth. The part of the plant most influenced also varied, with some strains inducing more roots, mainly length, and others inducing shoots. It has been reported that plants subjected to drought tend to increase root length in order to reach deeper layers of the soil where moisture is higher [34]. One of the strains (E7) significantly increased root length, increasing the ability to uptake water and adapt plants to drought. Root weight appeared to be inversely correlated with root length, highlighting the allocation of resources for elongation at the expense of the diameter and branching of the root system. This effect appeared to be more evident in plants inoculated with encapsulated bacteria than in plants directly exposed to bacteria. In fact, an increase in root length was reported in encapsulation trials, where studies demonstrated that the application of PGPR increased 29.7% to 100% maize roots [35,36].

The influence of the encapsulated bacteria was higher in the shoots than in the roots. The length and weight of shoots were higher in drought plants inoculated with all the encapsulated bacteria than in the non-inoculated control, three of which showed significant increases. The increase in weight was more significant, with one of the strains (F3) increasing in weight by 23%. Few studies have reported the impact of inoculated bacteria on shoot weight, but Minaxi and Saxena [37] demonstrated that encapsulated bacterial cells improved shoot growth compared with free inoculated cells. The present study also showed that drought influenced plant morphometry, with longer and less branched root systems and smaller shoots. Inoculation with certain strains (for example E7) accentuates this influence, and others (e.g., OS5-33, F3) minimized this effect.

It is well known that drought reduces the water potential of plant tissues. This decrease affects the solubility of biomolecules and the interference of ions (which become more concentrated) with cellular molecules and processes, causing the misfunction and generation of reactive oxygen species (ROS). ROS can react with important metabolic and physiological biomolecules causing irreversible damage (e.g., lipids and proteins) [38] and interfere negatively with cellular processes in membranes (photosynthesis and respiration) and cytosol (biochemical pathways). Organisms deal with oxidative stress through different mechanisms that scavenge ROS such as antioxidant compounds and enzymes [39].

The presence of alginate (capsules without bacteria) reduced the effects of drought (higher root and shoot growth), possibly due to the alginate composition of the capsules (polysaccharide composed of combinations of mannuronic/guluronic acids) that can retain water and, thus, may alleviate drought [40,41]. At the biochemical level, capsules did not reduce cellular damage; in fact, PC levels increased, but antioxidant protection (CAT) increased, and the decrease in proline compared to DC was compensated by the increase in carbohydrates, possibly due to the partial dissolution of the alginate capsules. Alginate is a polysaccharide, and the products of its degradation have already been reported to increase root and shoot length and seed germination [42,43]. The influence of encapsulated bacteria on the biochemistry of roots varied among strains. F3 and FS4-14 increased proline and soluble proteins, showing that the strategy to tolerate drought involved protecting cytosolic components and adapting cell metabolism to the new prevailing conditions induced by drought. Proline is an osmolyte that regulates cell water potential, acts as a molecular chaperone, stabilizes protein conformation and function, and helps detoxify reactive oxygen species [44]. Several studies have reported an association between proline and plant stress tolerance [26,38,45,46,47,48]. This study confirmed this protection, with most strains showing decreased protein carbonylation. However, increased proline failed to protect root membranes from high levels of LPO. Higher LPO levels were observed in the roots of maize plants inoculated with *Rhizobium* sp. [49]. O2-7 promoted ETS activity, which indicates a higher ability of root cells to produce energy (ATP) that can be used to induce mechanisms for cells to adapt to conditions imposed by drought. In fact, CAT activity was high in roots inoculated with O2-7, allowing the scavenging of hydrogen peroxide (H_2_O_2_) and decreasing lipid peroxidation. However, carbonylation was high, indicating that the protection of root cell membranes was effective, whereas the protection of proteins was not. This strain also increases sugars, which, in combination with proline, can regulate cells osmotically. The biochemical changes induced by E7 in root cells brought them closer to the root cells of the watered plants (Figure 4H). This strain was the one that most protected root cells from oxidative damage (lower LPO and PC), with low CAT activity and low sugar and proline levels, but high ETS activity, which was effective from a biochemical point of view, because root length was increased.

In the shoot, drought (DC) strongly decreased photosynthetic pigments (nearly 50%) and increased CAT activity but without a major impact on the other parameters analyzed. The capsules without bacteria had a smaller effect on the biochemistry of the shoots than the roots. However, the effect of the encapsulated bacteria was higher in the shoots than in the roots. Half or more of the strains significantly increased photosynthetic pigments to values close to those of the irrigated plants. Six strains showed increased protein and ETS levels relative to those in plants without capsules (WC and DC). CAT also increased, but only significantly, in one strain. However, the effects varied among strains. The influence on photosynthetic pigments was mainly from E7, F4-3, IX2-1, and D8. The increase in the ETS and protein levels was more evident in OS5-33, O2-7, F4-3, and E7. F3 had the greatest influence on CAT. The FS4-14 strain had a lower impact on shoot biochemistry (Figure 4H). Similar effects have also been reported by other authors, where Lopes et al. [49] reported that, in drought maize plants, inoculation with *Rhizobium* increased the levels of proline and carotenoids but did not alter the activity of antioxidant enzymes and the ETS or cell damage. Also, Cruz et al. [26], in drought maize plants inoculated with several PGPB strains, found contrasting responses among strains, with the majority promoting the production of proline, reducing cell damage (LPO and PC), the activity of antioxidant enzymes and the ETS, and few promoting sugar production and protein levels [50,51,52].

## 4. Material and Methods

### 4.1. Bacterial Strains

Bacteria were isolated from the roots of different plants (*Zea mays*, *Acacia albida*, *Tetraena simplex*, *Tetraena stapffii* and *Stipagrostis* sp.) harvested from arid and semi-arid environments. *Tetraena simplex*, *stapffii* and *Stipagrostis* sp. were isolated from the Namib desert (15°08′06.2″ S–12°12′51.7″ E) and *Zea mays* from Coruche, Portugal (38°56′41.637″ N–8°30′44.554″ W). Plant material was collected from a depth of 10–20 cm using a spade. Soil was carefully removed to preserve secondary roots, and samples were transported in an icebox. In the laboratory, samples were used immediately or stored at 4 °C for bacterial isolation. Roots were placed on yeast mannitol agar (YMA) plates and incubated at 26 °C. Colony growth was monitored twice daily, and isolates were subcultured to obtain pure cultures [49].

Bacterial strains were isolated from the Namib desert in collaboration with Sociedade Agrícola da Mumba, S.A. Company (Cuando Cubango, Angola) and identified by 16S rRNA gene sequencing in a previous publication [49] according with the methodology described by Cardoso et al. [53]. The partial 16S rRNA gene sequences from the representative isolates are available in GenBank (see Supplementary Table S1).

### 4.2. Bacteria Characterization

#### 4.2.1. Osmotolerance

Bacterial osmotolerance was assessed by growing bacteria in 5 mL tubes of yeast mannitol broth (YMB) [54] supplemented with different concentrations of polyethylene glycol 6000 (PEG): 5, 10, 12, 13, 14, 15, and 20%. The inoculated tubes were grown for 2 days at 26 °C in an orbital shaker (180 rpm) at 26 °C. Five replicates for each strain and concentration were used. Optical density was measured at 600 nm, and the results were used to calculate the IC_50_ (concentration that inhibits 50% bacterial growth) [55].

#### 4.2.2. Plant Growth Promotion (PGP) Traits

Siderophore production was quantified in accordance with the methodology described by Alexander and Zuberer [56]. The assessment of phosphate solubilization was conducted in accordance with the methodology described by Chatli et al. [57], and the methodology outlined by Meena et al. [58] was assessed for the evaluation of potassium solubilization. The results for siderophore, phosphate, and potassium solubilization capacities were expressed as the ratio of halo diameter to colony diameter (SI = halo diameter/colony diameter). Each strain was tested in triplicate.

The production of indole-3-acetic acid (IAA) was evaluated using the method described by Gordon and Weber [59] under conditions of osmotic stress (10% PEG). Alginate production was also measured under osmotic stress (10% PEG) stress in accordance with the methodology described by Johnson et al. [60]. Proline production was evaluated using the method described by Bates et al. [61], also under osmotic stress (10% PEG). The results are presented in micrograms of IAA, alginate, and proline per million cells (μg/M cells), with five replicates per strain.

### 4.3. Plant Growth Promotion Under Drought Stress (Experiment 1)

To evaluate the plant response to bacterial inoculation under drought conditions, bacteria with an IC_50_ higher than 15% PEG and high performance for plant growth-promoting (PGP) traits were selected for plant growth promotion testing under drought conditions (35% water holding capacity (WHC)). In each cup, one maize seed (Aquamax P9911, Pioneer, IA, USA) was sown in artificial soil (85% sand, 10% white clay, 5% peat, *w*/*w* ratio) and inoculated with 1 mL of bacterial inoculum (10^8^ cells/mL). Two uninoculated controls were used: watered (60% WHC) and drought (35% WHC). Plants were cultivated for two weeks in a greenhouse chamber under a 16 h light photoperiod (1450 μmol/m^2^/s) with temperatures maintained at 26 ± 1 °C during the day and 18 ± 1 °C at night. At the conclusion of the experiment, plants were harvested for measurements of root and shoot lengths as well as weight assessments. The bacterial strains that produced the most favorable results were chosen for encapsulation. The plants were cultivated for a period of two weeks in a greenhouse chamber that was set to a 16 h light cycle (1450 μmol/m^2^/s) at 26 ± 1 °C and 18 ± 1 °C. Upon completion of the experiment, measurements were taken of the root and shoot lengths, as well as the weight of the plants. The bacterial strains that yielded the most favorable outcomes were selected for encapsulation.

### 4.4. Bacteria Encapsulation

The encapsulation of bacterial strains was conducted using a sodium alginate–starch mixture via an extrusion technique [62,63], with some modifications. The starch (Stch) and alginate (CAS 9005-38-3 Sigma-Aldrich, St. Louis, MO, USA) (Alg) were dissolved by heating in a water bath until the solution reached boiling point. Once complete dissolution had occurred, the solutions were mixed to create a final alginate–starch solution. Bacterial cultures that had been grown overnight in YMB were subjected to centrifugation at 5000× *g* for 5 min. They were then resuspended in phosphate-buffered saline (PBS) and adjusted to a concentration of 10⁹ cells/mL. The bacterial suspension was then combined with the Alg-Stch solution at a 1:100 ratio (*v*:*v*), and capsules were produced by extrusion into a 5% calcium chloride (CaCl_2_) solution with continuous agitation for 1 h. The resulting capsules were then collected and incubated at 34 °C for 48 h to dry. The finished capsules were stored in sterile, sealed glass containers until required.

### 4.5. Plant Exposure to Encapsulated Bacteria (Experiment 2)

The above methodology was used to evaluate the effects of encapsulated bacteria on the growth of maize under drought stress (Section 4.3). Maize seedlings were sown and mixed with 100 mg of inoculated capsules containing previously selected bacteria. The same WHC was used, but two new control conditions were added: watered control (60% WHC) + capsules: non-inoculated capsules + 35% WHC. After two weeks, plants were collected for root and shoot measurements and stored at −80 °C for further biochemical analysis or used immediately for photosynthetic pigment measurements.

### 4.6. Photosynthetic Pigments

Fresh shoot samples were ground in cold 80% acetone as described in [64]. Chlorophyll a and b and carotenoid content were calculated according to the formulas described in Zhao et al. [65].

### 4.7. Biochemical Analysis

Proline determination:

Frozen samples were ground with a mortar and pestle in liquid nitrogen and then homogenized in 3% sulfosalicylic acid using an ultrasonic probe set to 0.6 Hz for 30 s [26]. The proline content was assessed using the method described by Bates et al. [61].

Extraction for other biochemical assays:

For the electron transport system (ETS), superoxide dismutase (SOD), catalase (CAT) activities and for protein (Prot) and protein carbonylation (PC) levels, frozen samples were initially ground with a mortar and pestle in liquid nitrogen and then homogenized in sodium phosphate buffer (50 mM of KH₂PO₄, 50 mM of K₂HPO₄, 1 mM of EDTA, 1% (*v*/*v*) Triton X-100, 1 mM of DTT, pH 7.0) at a 1:5 (*w*/*v*) ratio, as described in Lopes et al. [61]. Supernatant was employed in the assessment of ETS activity as described in King and Packard [66], with modifications by De Coen and Janssen [67]; Prot content was assessed by the method described by Robinson and Hogden [68]; SOD activity was determined by the method described by Beauchamp and Fridovich [69]; CAT activity was assessed by the method described by Johansson and Borg [70]; and PC levels was measured by the method described by Mesquita et al. [71]. The pellet was used to quantify lipid peroxidation (LPO) levels by the method described by Buege and Aust [72].

For sugar and starch content, samples were ground with a mortar and pestle in liquid nitrogen, and quantification was performed as described in Lopes et al. [64] following the method described by DuBois et al. [73].

### 4.8. Statistical and Multivariate Analysis

A one-factor, nonparametric permutational analysis of variance (PERMANOVA) was employed to test the hypothesis regarding the effects of encapsulated bacteria on photosynthetic pigments and biochemical parameters. The analysis was conducted using the PRIMER v7 software with the PERMANOVA+ add-on [74]. The dataset was subjected to a square root transformation, and a similarity matrix was constructed using a Euclidean distance metric. This was followed by a Monte Carlo test with 9999 permutations. Significance in pairwise comparisons was assessed with a *p*-value threshold of <0.05, with significant differences marked by an asterisk (*) in figures and tables. The null hypothesis proposed no differences between non-inoculated drought-stressed plants and other groups (watered plants and bacteria-encapsulated drought-stressed plants).

To analyze biochemical changes induced by encapsulated bacteria in drought-stressed plants, a Principal Coordinate Ordination (PCO) analysis was applied to data from photosynthetic pigments and biochemical assays. After performing a square root transformation, a Euclidean matrix was created for the PCO. Pearson correlation vectors (correlation ≥ 0.7) were then added to the PCO plot to identify the parameters most strongly impacting plant biochemistry across conditions.

A heatmap illustrating the plant growth-promoting (PGP) traits exhibited by each bacterial strain was constructed using MetaboAnalyst 5.0 [75]. The data were median-normalized for each condition and autoscaled.

## 5. Conclusions

This study demonstrates the potential of bacterial encapsulation as a strategy to enhance the resilience of young maize plants to drought stress. Biochemical analyses revealed that encapsulated bacteria modulated oxidative stress in whole plant cells, with specific strains reducing lipid peroxidation and protein carbonylation, thereby mitigating drought-induced cellular damage. Furthermore, the presence of inoculated capsules enhanced metabolic activity, as indicated by increased electron transport system activity and higher starch and sugar accumulation in roots. These metabolic changes suggest an improved energy balance and osmotic adjustment, both of which are essential for plant adaptation to drought conditions. In shoots, encapsulated bacteria significantly increased photosynthetic pigment concentrations, indicating a positive effect on photosynthetic efficiency under drought stress. The modulation of antioxidant enzyme activity, particularly catalase, further highlights the role of encapsulated bacteria in alleviating drought-induced oxidative stress. Collectively, these findings underscore the viability of bacterial encapsulation as a promising strategy to enhance crop resilience, reduce dependency on irrigation, and boost agricultural productivity in the context of escalating climate uncertainty. Subsequent studies should focus on long-term field applications and the potential synergies between encapsulated bacteria and other sustainable agricultural practices.

## Data Availability

The original contributions presented in this study are included in the article/Supplementary Material. Further inquiries can be directed to the corresponding author.

## Supplementary Materials

The following supporting information can be downloaded at https://www.mdpi.com/article/10.3390/plants14050812/s1, Supplementary Table S1: Identification by 16S rRNA gene sequencing of bacteria isolated from different plant roots (*Stipagrostis* sp., *Tetraena simplex* and *stapffii*, and *Zea mays*), selected for PGP traits assessment.
