# Supplementary material for: Inducing Drought Resilience in Maize Through Encapsulated Bacteria: Physiological and Biochemical Adaptations
Source: Plants (Basel). 2025 Mar 5;14(5):812. doi: 10.3390/plants14050812 (PMC11902389; doi:10.3390/plants14050812)
Supplement: Supplementary file 1 [file plants-14-00812-s001.zip › plants-3459050-supplementary.pdf]

Supplementary Table S1. Identification by 16S rRNA gene sequencing of bacteria isolated from different plant roots (*Stipagrostis* sp., *Tetraena simplex* and *stapffii*, and *Zea mays*), selected for PGP traits assessement.

Strain	Host species	Identification	Assession number	Site	Coordinates	Country	Soil use	Bioclimate
A1	*Stipagrostis* sp.	Unknown	-	Namibe Desert	15° 08' 6.2" S - 12° 12' 51.7" E	Angola	Natural	Hyperarid - inferior mesotropical
A2	*Stipagrostis* sp.	Unknown	-	Namibe Desert	15° 08' 6.2" S - 12° 12' 51.7" E	Angola	Natural	Hyperarid - inferior mesotropical
A3	*Stipagrostis* sp.	*Pseudarthrobacter*	PQ201021.1	Namibe Desert	15° 08' 6.2" S - 12° 12' 51.7" E	Angola	Natural	Hyperarid - inferior mesotropical
A4	*Stipagrostis* sp.	*Siccibacter*	PQ201022.1	Namibe Desert	15° 08' 6.2" S - 12° 12' 51.7" E	Angola	Natural	Hyperarid - inferior mesotropical
A5	*Stipagrostis* sp.	*Microbacterium*	PQ201023.1	Namibe Desert	15° 08' 6.2" S - 12° 12' 51.7" E	Angola	Natural	Hyperarid - inferior mesotropical
A6	*Stipagrostis* sp.	Unknown	-	Namibe Desert	15° 08' 6.2" S - 12° 12' 51.7" E	Angola	Natural	Hyperarid - inferior mesotropical
A7	*Stipagrostis* sp.	*Pseudomonas*	PQ201024.1	Namibe Desert	15° 08' 6.2" S - 12° 12' 51.7" E	Angola	Natural	Hyperarid - inferior mesotropical
A8	*Stipagrostis* sp.	Unknown	-	Namibe Desert	15° 08' 6.2" S - 12° 12' 51.7" E	Angola	Natural	Hyperarid - inferior mesotropical
A9	*Stipagrostis* sp.	*Pseudomonas*	PQ201025.1	Namibe Desert	15° 08' 6.2" S - 12° 12' 51.7" E	Angola	Natural	Hyperarid - inferior mesotropical
A10	*Stipagrostis* sp.	Unknown	-	Namibe Desert	15° 08' 6.2" S - 12° 12' 51.7" E	Angola	Natural	Hyperarid - inferior mesotropical
A11	*Stipagrostis* sp.	Unknown	-	Namibe Desert	15° 08' 6.2" S - 12° 12' 51.7" E	Angola	Natural	Hyperarid - inferior mesotropical
A12	*Stipagrostis* sp.	*Pseudomonas*	PQ201026.1	Namibe Desert	15° 08' 6.2" S - 12° 12' 51.7" E	Angola	Natural	Hyperarid - inferior mesotropical
A13	*Stipagrostis* sp.	*Acinetobacter*	PQ201027.1	Namibe Desert	15° 08' 6.2" S - 12° 12' 51.7" E	Angola	Natural	Hyperarid - inferior mesotropical
A14	*Stipagrostis* sp.	*Stenotrophomonas*	PQ201028.1	Namibe Desert	15° 08' 6.2" S - 12° 12' 51.7" E	Angola	Natural	Hyperarid - inferior mesotropical
A15	*Stipagrostis* sp.	*Cronobacter*	PQ201029.1	Namibe Desert	15° 08' 6.2" S - 12° 12' 51.7" E	Angola	Natural	Hyperarid - inferior mesotropical
A16	*Stipagrostis* sp.	*Enterobacter*	PQ201030.1	Namibe Desert	15° 08' 6.2" S - 12° 12' 51.7" E	Angola	Natural	Hyperarid - inferior mesotropical
A17	*Stipagrostis* sp.	Unknown	-	Namibe Desert	15° 08' 6.2" S - 12° 12' 51.7" E	Angola	Natural	Hyperarid - inferior mesotropical
A18	*Stipagrostis* sp.	Unknown	-	Namibe Desert	15° 08' 6.2" S - 12° 12' 51.7" E	Angola	Natural	Hyperarid - inferior mesotropical
B1	*Stipagrostis* sp.	*Siccibacter*	PQ201031.1	Namibe Desert	15° 08' 6.2" S - 12° 12' 51.7" E	Angola	Natural	Hyperarid - inferior mesotropical
B2	*Stipagrostis* sp.	*Siccibacter*	PQ201032.1	Namibe Desert	15° 08' 6.2" S - 12° 12' 51.7" E	Angola	Natural	Hyperarid - inferior mesotropical
B3	*Stipagrostis* sp.	Unknown	-	Namibe Desert	15° 08' 6.2" S - 12° 12' 51.7" E	Angola	Natural	Hyperarid - inferior mesotropical
B4	*Stipagrostis* sp.	*Enterobacter*	PQ201033.1	Namibe Desert	15° 08' 6.2" S - 12° 12' 51.7" E	Angola	Natural	Hyperarid - inferior mesotropical
B5	*Stipagrostis* sp.	Unknown	-	Namibe Desert	15° 08' 6.2" S - 12° 12' 51.7" E	Angola	Natural	Hyperarid - inferior mesotropical
B6	*Stipagrostis* sp.	Unknown	-	Namibe Desert	15° 08' 6.2" S - 12° 12' 51.7" E	Angola	Natural	Hyperarid - inferior mesotropical
B7	*Stipagrostis* sp.	*Pseudomonas*	PQ201034.1	Namibe Desert	15° 08' 6.2" S - 12° 12' 51.7" E	Angola	Natural	Hyperarid - inferior mesotropical
B8	*Stipagrostis* sp.	Unknown	-	Namibe Desert	15° 08' 6.2" S - 12° 12' 51.7" E	Angola	Natural	Hyperarid - inferior mesotropical
B9	*Stipagrostis* sp.	Unknown	-	Namibe Desert	15° 08' 6.2" S - 12° 12' 51.7" E	Angola	Natural	Hyperarid - inferior mesotropical
B10	*Stipagrostis* sp.	*Pantoea*	PQ201035.1	Namibe Desert	15° 08' 6.2" S - 12° 12' 51.7" E	Angola	Natural	Hyperarid - inferior mesotropical
B11	*Stipagrostis* sp.	*Pseudomonas*	PQ201036.1	Namibe Desert	15° 08' 6.2" S - 12° 12' 51.7" E	Angola	Natural	Hyperarid - inferior mesotropical
B12	*Stipagrostis* sp.	*Pseudomonas*	PQ201037.1	Namibe Desert	15° 08' 6.2" S - 12° 12' 51.7" E	Angola	Natural	Hyperarid - inferior mesotropical
B13	*Stipagrostis* sp.	Unknown	-	Namibe Desert	15° 08' 6.2" S - 12° 12' 51.7" E	Angola	Natural	Hyperarid - inferior mesotropical

B14	*Stipagrostis* sp.	Unknown	-	Namibe Desert	15° 08' 6.2" S - 12° 12' 51.7" E	Angola	Natural	Hyperarid - inferior mesotropical
B15	*Stipagrostis* sp.	*Microbacterium*	PQ201038.1	Namibe Desert	15° 08' 6.2" S - 12° 12' 51.7" E	Angola	Natural	Hyperarid - inferior mesotropical
B16	*Stipagrostis* sp.	*Enterobacter*	PQ201039.1	Namibe Desert	15° 08' 6.2" S - 12° 12' 51.7" E	Angola	Natural	Hyperarid - inferior mesotropical
B17	*Stipagrostis* sp.	Unknown	-	Namibe Desert	15° 08' 6.2" S - 12° 12' 51.7" E	Angola	Natural	Hyperarid - inferior mesotropical
B18	*Stipagrostis* sp.	*Enterobacter*	PQ201040.1	Namibe Desert	15° 08' 6.2" S - 12° 12' 51.7" E	Angola	Natural	Hyperarid - inferior mesotropical
B19	*Stipagrostis* sp.	*Caulobacter*	PQ201041.1	Namibe Desert	15° 08' 6.2" S - 12° 12' 51.7" E	Angola	Natural	Hyperarid - inferior mesotropical
B20	*Stipagrostis* sp.	*Acinetobacter*	PQ201042.1	Namibe Desert	15° 08' 6.2" S - 12° 12' 51.7" E	Angola	Natural	Hyperarid - inferior mesotropical
B21	*Stipagrostis* sp.	Unknown	-	Namibe Desert	15° 08' 6.2" S - 12° 12' 51.7" E	Angola	Natural	Hyperarid - inferior mesotropical
B22	*Stipagrostis* sp.	*Pseudomonas*	PQ201043.1	Namibe Desert	15° 08' 6.2" S - 12° 12' 51.7" E	Angola	Natural	Hyperarid - inferior mesotropical
B23	*Stipagrostis* sp.	*Priestia*	PQ201044.1	Namibe Desert	15° 08' 6.2" S - 12° 12' 51.7" E	Angola	Natural	Hyperarid - inferior mesotropical
C1	*Tetraena simplex* (L.) Beier & Thulin	*Flavobacterium*	PQ201045.1	Namibe Desert	15° 08' 6.2" S - 12° 12' 51.7" E	Angola	Natural	Hyperarid - inferior mesotropical
C2	*Tetraena simplex* (L.) Beier & Thulin	*Enterobacter*	PQ201046.1	Namibe Desert	15° 08' 6.2" S - 12° 12' 51.7" E	Angola	Natural	Hyperarid - inferior mesotropical
C3	*Tetraena simplex* (L.) Beier & Thulin	*Chryseobacterium*	PQ201047.1	Namibe Desert	15° 08' 6.2" S - 12° 12' 51.7" E	Angola	Natural	Hyperarid - inferior mesotropical
C4	*Tetraena simplex* (L.) Beier & Thulin	*Cronobacter*	PQ201048.1	Namibe Desert	15° 08' 6.2" S - 12° 12' 51.7" E	Angola	Natural	Hyperarid - inferior mesotropical
C5	*Tetraena simplex* (L.) Beier & Thulin	Unknown	-	Namibe Desert	15° 08' 6.2" S - 12° 12' 51.7" E	Angola	Natural	Hyperarid - inferior mesotropical
C6	*Tetraena simplex* (L.) Beier & Thulin	*Stenotrophomonas*	PQ201049.1	Namibe Desert	15° 08' 6.2" S - 12° 12' 51.7" E	Angola	Natural	Hyperarid - inferior mesotropical
C7	*Tetraena simplex* (L.) Beier & Thulin	Unknown	-	Namibe Desert	15° 08' 6.2" S - 12° 12' 51.7" E	Angola	Natural	Hyperarid - inferior mesotropical
C8	*Tetraena simplex* (L.) Beier & Thulin	Unknown	-	Namibe Desert	15° 08' 6.2" S - 12° 12' 51.7" E	Angola	Natural	Hyperarid - inferior mesotropical
C9	*Tetraena simplex* (L.) Beier & Thulin	Unknown	-	Namibe Desert	15° 08' 6.2" S - 12° 12' 51.7" E	Angola	Natural	Hyperarid - inferior mesotropical
C10	*Tetraena simplex* (L.) Beier & Thulin	*Aeromonas*	PQ201050.1	Namibe Desert	15° 08' 6.2" S - 12° 12' 51.7" E	Angola	Natural	Hyperarid - inferior mesotropical
C11	*Tetraena simplex* (L.) Beier & Thulin	Unknown	-	Namibe Desert	15° 08' 6.2" S - 12° 12' 51.7" E	Angola	Natural	Hyperarid - inferior mesotropical
C12	*Tetraena simplex* (L.) Beier & Thulin	*Paenibacillus*	PQ201051.1	Namibe Desert	15° 08' 6.2" S - 12° 12' 51.7" E	Angola	Natural	Hyperarid - inferior mesotropical
C13	*Tetraena simplex* (L.) Beier & Thulin	Unknown	-	Namibe Desert	15° 08' 6.2" S - 12° 12' 51.7" E	Angola	Natural	Hyperarid - inferior mesotropical
C14	*Tetraena simplex* (L.) Beier & Thulin	*Acinetobacter*	PQ201052.1	Namibe Desert	15° 08' 6.2" S - 12° 12' 51.7" E	Angola	Natural	Hyperarid - inferior mesotropical
C15	*Tetraena simplex* (L.) Beier & Thulin	Unknown	-	Namibe Desert	15° 08' 6.2" S - 12° 12' 51.7" E	Angola	Natural	Hyperarid - inferior mesotropical
C16	*Tetraena simplex* (L.) Beier & Thulin	*Xanthomonas*	PQ201053.1	Namibe Desert	15° 08' 6.2" S - 12° 12' 51.7" E	Angola	Natural	Hyperarid - inferior mesotropical
C17	*Tetraena simplex* (L.) Beier & Thulin	Unknown	-	Namibe Desert	15° 08' 6.2" S - 12° 12' 51.7" E	Angola	Natural	Hyperarid - inferior mesotropical
C18	*Tetraena simplex* (L.) Beier & Thulin	*Rhodococcus*	PQ201054.1	Namibe Desert	15° 08' 6.2" S - 12° 12' 51.7" E	Angola	Natural	Hyperarid - inferior mesotropical
C19	*Tetraena simplex* (L.) Beier & Thulin	*Pseudomonas*	PQ201055.1	Namibe Desert	15° 08' 6.2" S - 12° 12' 51.7" E	Angola	Natural	Hyperarid - inferior mesotropical
C20	*Tetraena simplex* (L.) Beier & Thulin	Unknown	-	Namibe Desert	15° 08' 6.2" S - 12° 12' 51.7" E	Angola	Natural	Hyperarid - inferior mesotropical
D1	*Tetraena simplex* (L.) Beier & Thulin	Unknown	-	Namibe Desert	15° 08' 6.2" S - 12° 12' 51.7" E	Angola	Natural	Hyperarid - inferior mesotropical
D2	*Tetraena simplex* (L.) Beier & Thulin	*Pseudomonas*	PQ201056.1	Namibe Desert	15° 08' 6.2" S - 12° 12' 51.7" E	Angola	Natural	Hyperarid - inferior mesotropical
D3	*Tetraena simplex* (L.) Beier & Thulin	*Enterobacter*	PQ201057.1	Namibe Desert	15° 08' 6.2" S - 12° 12' 51.7" E	Angola	Natural	Hyperarid - inferior mesotropical
D4	*Tetraena simplex* (L.) Beier & Thulin	*Pantoea*	PQ201058.1	Namibe Desert	15° 08' 6.2" S - 12° 12' 51.7" E	Angola	Natural	Hyperarid - inferior mesotropical

D5	*Tetraena simplex* (L.) Beier & Thulin	*Enterobacter*	PQ201059.1	Namibe Desert	15° 08' 6.2" S - 12° 12' 51.7" E	Angola	Natural	Hyperarid - inferior mesotropical
D6	*Tetraena simplex* (L.) Beier & Thulin	Unknown	-	Namibe Desert	15° 08' 6.2" S - 12° 12' 51.7" E	Angola	Natural	Hyperarid - inferior mesotropical
D7	*Tetraena simplex* (L.) Beier & Thulin	Unknown	-	Namibe Desert	15° 08' 6.2" S - 12° 12' 51.7" E	Angola	Natural	Hyperarid - inferior mesotropical
D8	*Tetraena simplex* (L.) Beier & Thulin	*Pseudomonas*	PQ201060	Namibe Desert	15° 08' 6.2" S - 12° 12' 51.7" E	Angola	Natural	Hyperarid - inferior mesotropical
D9	*Tetraena simplex* (L.) Beier & Thulin	Unknown	-	Namibe Desert	15° 08' 6.2" S - 12° 12' 51.7" E	Angola	Natural	Hyperarid - inferior mesotropical
D10	*Tetraena simplex* (L.) Beier & Thulin	Unknown	-	Namibe Desert	15° 08' 6.2" S - 12° 12' 51.7" E	Angola	Natural	Hyperarid - inferior mesotropical
D11	*Tetraena simplex* (L.) Beier & Thulin	*Stenotrophomonas*	PQ201061.1	Namibe Desert	15° 08' 6.2" S - 12° 12' 51.7" E	Angola	Natural	Hyperarid - inferior mesotropical
D12	*Tetraena simplex* (L.) Beier & Thulin	Unknown	-	Namibe Desert	15° 08' 6.2" S - 12° 12' 51.7" E	Angola	Natural	Hyperarid - inferior mesotropical
D13	*Tetraena simplex* (L.) Beier & Thulin	*Siccibacter*	PQ201062.1	Namibe Desert	15° 08' 6.2" S - 12° 12' 51.7" E	Angola	Natural	Hyperarid - inferior mesotropical
E1	*Tetraena stapfii* (Schinz) Beier & Thulin	*Bradyrhizobium*	PQ201063.1	Namibe Desert	15° 08' 6.2" S - 12° 12' 51.7" E	Angola	Natural	Hyperarid - inferior mesotropical
E2	*Tetraena stapfii* (Schinz) Beier & Thulin	Unknown	-	Namibe Desert	15° 08' 6.2" S - 12° 12' 51.7" E	Angola	Natural	Hyperarid - inferior mesotropical
E3	*Tetraena stapfii* (Schinz) Beier & Thulin	Unknown	-	Namibe Desert	15° 08' 6.2" S - 12° 12' 51.7" E	Angola	Natural	Hyperarid - inferior mesotropical
E4	*Tetraena stapfii* (Schinz) Beier & Thulin	Unknown	-	Namibe Desert	15° 08' 6.2" S - 12° 12' 51.7" E	Angola	Natural	Hyperarid - inferior mesotropical
E5	*Tetraena stapfii* (Schinz) Beier & Thulin	Unknown	-	Namibe Desert	15° 08' 6.2" S - 12° 12' 51.7" E	Angola	Natural	Hyperarid - inferior mesotropical
E6	*Tetraena stapfii* (Schinz) Beier & Thulin	*Pseudomonas*	PQ201064.1	Namibe Desert	15° 08' 6.2" S - 12° 12' 51.7" E	Angola	Natural	Hyperarid - inferior mesotropical
E7	*Tetraena stapfii* (Schinz) Beier & Thulin	Unknown	-	Namibe Desert	15° 08' 6.2" S - 12° 12' 51.7" E	Angola	Natural	Hyperarid - inferior mesotropical
E8	*Tetraena stapfii* (Schinz) Beier & Thulin	*Enterobacter*	PQ201065.1	Namibe Desert	15° 08' 6.2" S - 12° 12' 51.7" E	Angola	Natural	Hyperarid - inferior mesotropical
E9	*Tetraena stapfii* (Schinz) Beier & Thulin	Unknown	-	Namibe Desert	15° 08' 6.2" S - 12° 12' 51.7" E	Angola	Natural	Hyperarid - inferior mesotropical
E10	*Tetraena stapfii* (Schinz) Beier & Thulin	Unknown	-	Namibe Desert	15° 08' 6.2" S - 12° 12' 51.7" E	Angola	Natural	Hyperarid - inferior mesotropical
E11	*Tetraena stapfii* (Schinz) Beier & Thulin	Unknown	-	Namibe Desert	15° 08' 6.2" S - 12° 12' 51.7" E	Angola	Natural	Hyperarid - inferior mesotropical
F1	*Tetraena stapfii* (Schinz) Beier & Thulin	*Acinetobacter*	PQ201066.1	Namibe Desert	15° 08' 6.2" S - 12° 12' 51.7" E	Angola	Natural	Hyperarid - inferior mesotropical
F2	*Tetraena stapfii* (Schinz) Beier & Thulin	*Massilia*	PQ201067.1	Namibe Desert	15° 08' 6.2" S - 12° 12' 51.7" E	Angola	Natural	Hyperarid - inferior mesotropical
F3	*Tetraena stapfii* (Schinz) Beier & Thulin	*Rhizobium*	PQ201068	Namibe Desert	15° 08' 6.2" S - 12° 12' 51.7" E	Angola	Natural	Hyperarid - inferior mesotropical
F4	*Tetraena stapfii* (Schinz) Beier & Thulin	Unknown	-	Namibe Desert	15° 08' 6.2" S - 12° 12' 51.7" E	Angola	Natural	Hyperarid - inferior mesotropical
F5	*Tetraena stapfii* (Schinz) Beier & Thulin	*Stenotrophomonas*	PQ201069.1	Namibe Desert	15° 08' 6.2" S - 12° 12' 51.7" E	Angola	Natural	Hyperarid - inferior mesotropical
F6	*Tetraena stapfii* (Schinz) Beier & Thulin	Unknown	-	Namibe Desert	15° 08' 6.2" S - 12° 12' 51.7" E	Angola	Natural	Hyperarid - inferior mesotropical

F7	*Tetraena stapfii* (Schinz) Beier & Thulin	*Rhizobium*	PQ201070.1	Namibe Desert	15° 08' 6.2" S - 12° 12' 51.7" E	Angola	Natural	Hyperarid - inferior mesotropical
F8	*Tetraena stapfii* (Schinz) Beier & Thulin	*Pseudomonas*	PQ201071.1	Namibe Desert	15° 08' 6.2" S - 12° 12' 51.7" E	Angola	Natural	Hyperarid - inferior mesotropical
F9	*Tetraena stapfii* (Schinz) Beier & Thulin	*Pseudomonas*	PQ201072.1	Namibe Desert	15° 08' 6.2" S - 12° 12' 51.7" E	Angola	Natural	Hyperarid - inferior mesotropical
F10	*Tetraena stapfii* (Schinz) Beier & Thulin	Unknown	-	Namibe Desert	15° 08' 6.2" S - 12° 12' 51.7" E	Angola	Natural	Hyperarid - inferior mesotropical
F11	*Tetraena stapfii* (Schinz) Beier & Thulin	Siccibacter	PQ201073	Namibe Desert	15° 08' 6.2" S - 12° 12' 51.7" E	Angola	Natural	Hyperarid - inferior mesotropical
F12	*Tetraena stapfii* (Schinz) Beier & Thulin	*Pseudomonas*	PQ201074.1	Namibe Desert	15° 08' 6.2" S - 12° 12' 51.7" E	Angola	Natural	Hyperarid - inferior mesotropical
F13	*Tetraena stapfii* (Schinz) Beier & Thulin	*Pseudomonas*	PQ201075.1	Namibe Desert	15° 08' 6.2" S - 12° 12' 51.7" E	Angola	Natural	Hyperarid - inferior mesotropical
F14	*Tetraena stapfii* (Schinz) Beier & Thulin	Unknown	-	Namibe Desert	15° 08' 6.2" S - 12° 12' 51.7" E	Angola	Natural	Hyperarid - inferior mesotropical
FX5-23	*Zea mays*	*Pseudomonas*	OR948253.1	Coruche	16° 45' 15" N - 22° 56' 45" W	Portugal	Agricultural	Inferior dry - superior thermomediterranean
IC4-20	*Zea mays*	Unknown	-	Coruche	16° 45' 15" N - 22° 56' 45" W	Portugal	Agricultural	Inferior dry - superior thermomediterranean
IS2-11	*Zea mays*	Unknown	-	Coruche	16° 45' 15" N - 22° 56' 45" W	Portugal	Agricultural	Inferior dry - superior thermomediterranean
IS3-2	*Zea mays*	Unknown	-	Coruche	16° 45' 15" N - 22° 56' 45" W	Portugal	Agricultural	Inferior dry - superior thermomediterranean
IS4-21	*Zea mays*	Unknown	-	Coruche	16° 45' 15" N - 22° 56' 45" W	Portugal	Agricultural	Inferior dry - superior thermomediterranean
O1-35	*Zea mays*	Unknown	-	Coruche	16° 45' 15" N - 22° 56' 45" W	Portugal	Agricultural	Inferior dry - superior thermomediterranean
C1-13	*Zea mays*	Unknown	-	Coruche	16° 45' 15" N - 22° 56' 45" W	Portugal	Agricultural	Inferior dry - superior thermomediterranean
F1-21	*Zea mays*	Unknown	-	Coruche	16° 45' 15" N - 22° 56' 45" W	Portugal	Agricultural	Inferior dry - superior thermomediterranean
F1-35	*Zea mays*	Unknown	-	Coruche	16° 45' 15" N - 22° 56' 45" W	Portugal	Agricultural	Inferior dry - superior thermomediterranean
F4-3	*Zea mays*	Unknown	-	Coruche	16° 45' 15" N - 22° 56' 45" W	Portugal	Agricultural	Inferior dry - superior thermomediterranean
FS3-11	*Zea mays*	Unknown	-	Coruche	16° 45' 15" N - 22° 56' 45" W	Portugal	Agricultural	Inferior dry - superior thermomediterranean
FS4-14	*Zea mays*	Acinetobacter	OR948275.1	Coruche	16° 45' 15" N - 22° 56' 45" W	Portugal	Agricultural	Inferior dry - superior thermomediterranean
FX5-23	*Zea mays*	*Pseudomonas*	OR948253.1	Coruche	16° 45' 15" N - 22° 56' 45" W	Portugal	Agricultural	Inferior dry - superior thermomediterranean
IC4-20	*Zea mays*	Unknown	-	Coruche	16° 45' 15" N - 22° 56' 45" W	Portugal	Agricultural	Inferior dry - superior thermomediterranean
IC4-21	*Zea mays*	*Pseudomonas*	OR948152.1	Coruche	16° 45' 15" N - 22° 56' 45" W	Portugal	Agricultural	Inferior dry - superior thermomediterranean
IS2-11	*Zea mays*	Unknown	-	Coruche	16° 45' 15" N - 22° 56' 45" W	Portugal	Agricultural	Inferior dry - superior thermomediterranean
IS3-2	*Zea mays*	Unknown	-	Coruche	16° 45' 15" N - 22° 56' 45" W	Portugal	Agricultural	Inferior dry - superior thermomediterranean
IS4-21	*Zea mays*	Unknown	-	Coruche	16° 45' 15" N - 22° 56' 45" W	Portugal	Agricultural	Inferior dry - superior thermomediterranean
IX2-1	*Zea mays*	*Pantoea*	OR948171.1	Coruche	16° 45' 15" N - 22° 56' 45" W	Portugal	Agricultural	Inferior dry - superior thermomediterranean
O1-35	*Zea mays*	Unknown	-	Coruche	16° 45' 15" N - 22° 56' 45" W	Portugal	Agricultural	Inferior dry - superior thermomediterranean
O2-20	*Zea mays*	Unknown	-	Coruche	16° 45' 15" N - 22° 56' 45" W	Portugal	Agricultural	Inferior dry - superior thermomediterranean
O2-7	*Zea mays*	Unknown	-	Coruche	16° 45' 15" N - 22° 56' 45" W	Portugal	Agricultural	Inferior dry - superior thermomediterranean

O3-3	*Zea mays*	Unknown	-	Coruche	16° 45' 15" N - 22° 56' 45" W	Portugal	Agricultural	Inferior dry - superior thermomediterranean
OS4-2	*Zea mays*	*Pseudomonas*	OR948322.1	Coruche	16° 45' 15" N - 22° 56' 45" W	Portugal	Agricultural	Inferior dry - superior thermomediterranean
OS5-33	*Zea mays*	*Pseudomonas*	OR948111.1	Coruche	16° 45' 15" N - 22° 56' 45" W	Portugal	Agricultural	Inferior dry - superior thermomediterranean
OX2-19	*Zea mays*	*Cedecea*	OR948089.1	Coruche	16° 45' 15" N - 22° 56' 45" W	Portugal	Agricultural	Inferior dry - superior thermomediterranean
OX3-23	*Zea mays*	*Burkholderia*	OR948363.1	Coruche	16° 45' 15" N - 22° 56' 45" W	Portugal	Agricultural	Inferior dry - superior thermomediterranean
